# Unraveling Stigmas of Male Breast Carcinoma: A Singular Case of Advanced Metaplastic Breast Carcinoma in a Young Male

**DOI:** 10.7759/cureus.44174

**Published:** 2023-08-26

**Authors:** Muhammad Awais Kanwal, Umaisa Khalid, Rafiya Ali Athar, Muhammad Asad Parvaiz, Mohammad Zulqarnain Chaudhry

**Affiliations:** 1 Surgical Oncology, Shaukat Khanum Memorial Cancer Hospital and Research Centre, Lahore, PAK

**Keywords:** malignancy of breast cancer in males, breast carcinoma in males, stigmas of male breast cancer, variant of metaplastic breast cancer, metastatic metaplastic breast cancer, oncology, male breast cancers

## Abstract

Breast carcinoma in males is a rare and unique condition that differs from breast cancer in females and is typically diagnosed at an advanced stage in older male patients. Late diagnosis is often due to the rarity of male breast carcinoma. Among the various types of breast carcinomas, metaplastic breast carcinoma is one of the rarest kind of breast malignancy and is associated with poorer outcomes. This case report presents a singular case of a young male in his early thirties who presented with a breast lump and was diagnosed with metaplastic breast carcinoma. Breast cancer in males is a topic that is often overlooked and lacks extensive research. However, with an increasing incidence of breast carcinoma in males, including even the rarest forms, such as metaplastic carcinoma, and its occurrence in young patients as highlighted in this case report, it is crucial to initiate more discussions, enhance education, and promote further research in male breast carcinoma. In addition, the psychosocial impacts of the disease should be carefully considered, as men with breast cancer face unique emotional challenges that require attention and support.

## Introduction

Breast carcinoma in males is an uncommon and distinct condition that presents notable differences compared to breast cancer in females. While breast cancer is widely recognized as a prevalent disease affecting women, it is often overlooked that it can also affect men. Accounting for less than 1% of all breast carcinomas, breast cancer in males presents a unique set of challenges in terms of diagnosis, treatment, and understanding [1].

Unlike breast cancer in women, breast carcinoma in males is typically diagnosed at an advanced stage and often in older male patients. Late diagnosis is primarily attributed to the rarity of male breast carcinoma, which contributes to a lack of awareness among healthcare providers and delays in seeking medical attention. This delay in diagnosis can have significant implications for the prognosis and treatment options available to male patients [2,3].

Among the various types of breast carcinomas, metaplastic breast carcinoma is an exceptionally rare subtype. Metaplastic breast carcinoma is characterized by its distinct histological features, exhibiting a mixture of epithelial and mesenchymal components. It accounts for less than 1% of all breast malignancies and is associated with poorer outcomes compared to other breast cancer subtypes [4,5].

## Case presentation

**Figure 1:**
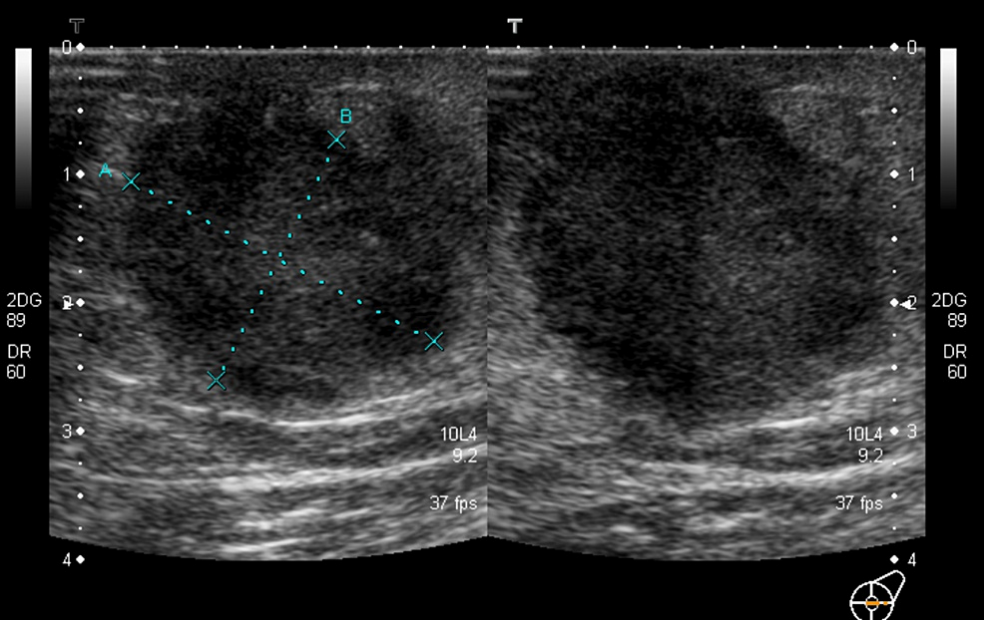
Ultrasound breast done as a part of the triple assessment The hypoechoic lobulated mass measured 27 x 21 mm with internal specks of calcification and increased posterior accentuation in the left retroareolar region. Imaging features were consistent with primary malignant breast lesion. No other lesions in the left breast were seen. Suspicious-appearing lymph nodes were seen in the left axilla, with the largest measuring 26 x 17 mm.

A young male in his early thirties presented with a lump in his left breast that had been present for more than six months. On examination, the right breast appeared normal, but the left breast had a 3 x 3 cm deep-seated lump in the retroareolar region. The lump was not attached to the skin or muscles. Enlarged lymph nodes were palpable in the left axilla. To further assess the condition, a triple assessment was conducted. Ultrasound of the breast (Figure 1) revealed a 27 x 21 mm mass in the retroareolar region and suspicious lymph nodes in the left axilla.

**Figure 2:**
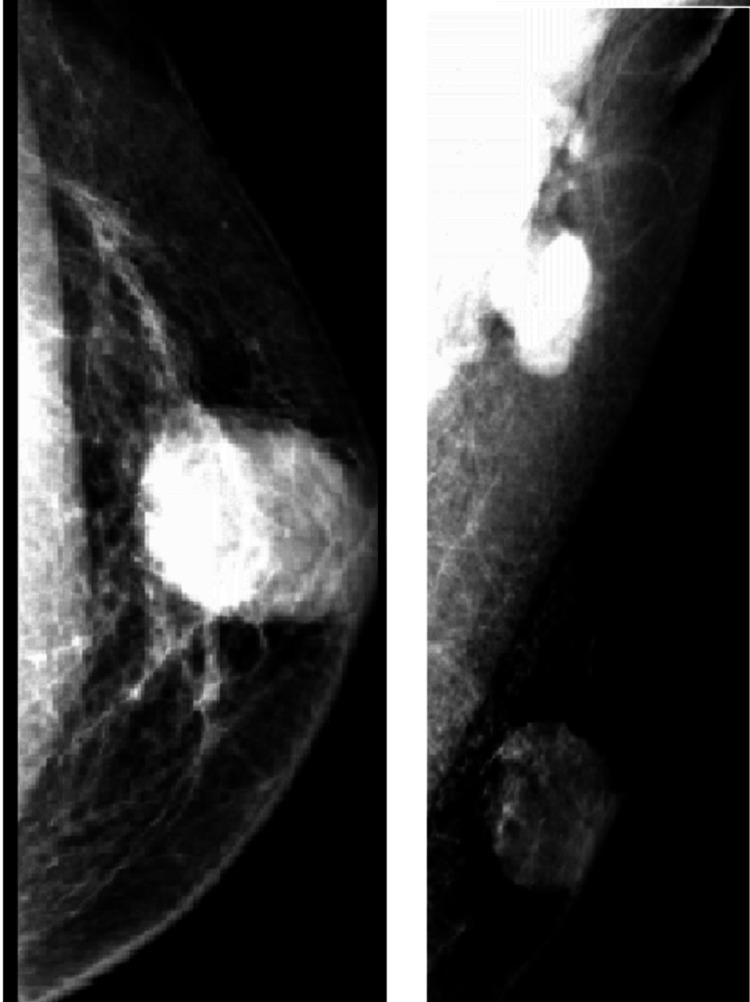
Mammogram showing high-density circumscribed opacity in the left retroareolar compartment associated with few microcalcifications concerning for a primary breast malignant mass. No satellite nodules were seen. The left axilla demonstrated large lobulated lymph nodes.

Mammography (Figure 2) was also performed, which showed a high-density circumscribed opacity in the left retroareolar compartment, along with a few microcalcifications.

A core biopsy of the left breast lump was performed under ultrasound guidance, and it revealed invasive ductal carcinoma with metaplastic features, classified as grade II. Fine needle aspiration of the left axillary lymph node confirmed metastatic adenocarcinoma. Immunohistochemistry stains were conducted, and the results are presented in Table 1.

**Table 1:** Immunohistochemical staining results done on the initial tissue biopsy HER-2/neu, human epidermal growth factor receptor 2

Estrogen receptors	Strongly positive, nuclear staining 80-90% tumor cells
Progesterone receptors	Strongly positive, nuclear staining 80-90% tumor cells
HER-2/neu	Equivocal (2+)
Ki-67	Positive in 25-30% tumor cells

**Figure 3:**
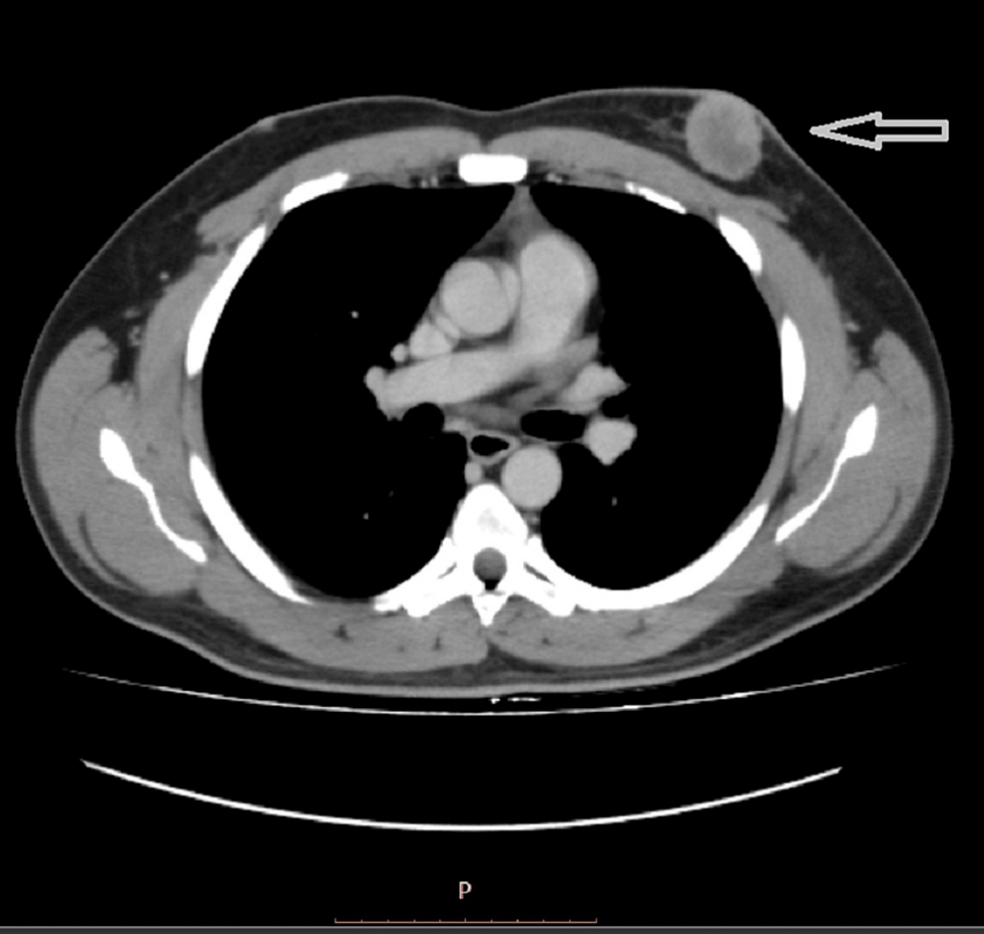
Computed tomography for the metastatic workup The left breast demonstrated a large, enhancing circumscribed retroareolar mass measuring 3.4 cm in maximum dimension associated with ipsilateral metastatic axillary node, with the largest having a short axis diameter of 2.5 cm. There was a plump benign-appearing subcarinal lymph node with a short axis diameter of 1.1 cm along with a right hilar subcentimeter calcified node. The visualized lower cervical and the right axilla and supraclavicular recesses were clear. There were no suspicious pulmonary or pleural focal abnormality or effusions. There was no evidence of hepatopulmonary or osseous metastasis.

To assess the extent of metastasis, a computed tomography (CT) scan was performed (Figure 3), which revealed a primary mass in the left breast with ipsilateral axillary lymph node involvement. No evidence of metastasis to the liver, lungs, or bones was found.

The case was discussed in a multidisciplinary team (MDT) meeting, and the recommendation was to administer neoadjuvant chemotherapy followed by a modified radical mastectomy. The patient received four cycles of doxorubicin (A) and cyclophosphamide (C), followed by four cycles of paclitaxel. The patient tolerated the chemotherapy well with minimal side effects.

**Figure 4:**
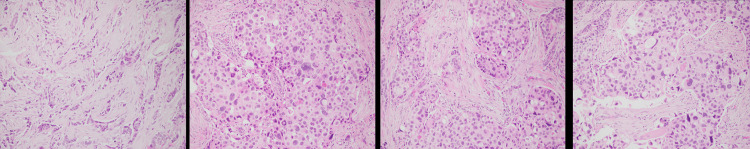
Microscopic evaluation Sections revealed breast parenchyma with an infiltrating tumor composed of nests, irregular cords, and trabeculae. The cells had an abundant cytoplasm and displayed marked nuclear pleomorphism and distinct nucleoli. Areas of squamous differentiation with keratinization and dyskeratotic cells were also seen. Fibrous-appearing stroma was present.

After completing chemotherapy, the patient underwent a left modified radical mastectomy. The histopathology report (Figure 4) indicated a 2.2 cm invasive ductal carcinoma with metaplastic features, graded as grade III. The tumor invaded the dermis without skin ulceration. The resection margins were free of tumor, and none of the 13 lymph nodes examined showed evidence of tumor.

Microscopic examination of the breast parenchyma revealed an infiltrating tumor composed of nests, cords, and trabeculae with limited stroma. The tumor exhibited marked nuclear pleomorphism, prominent nucleoli, and brisk mitotic activity. There were also squamous areas of the tumor showing markedly pleomorphic cells with a high nucleus-to-cytoplasm ratio and abundant eosinophilic cytoplasm.

Following surgery, radiation therapy was administered to the left chest wall and left supraclavicular fossa. The patient was also started on tamoxifen for hormonal therapy. The patient had regular follow-ups and remained in remission for three years.

**Figure 5:**
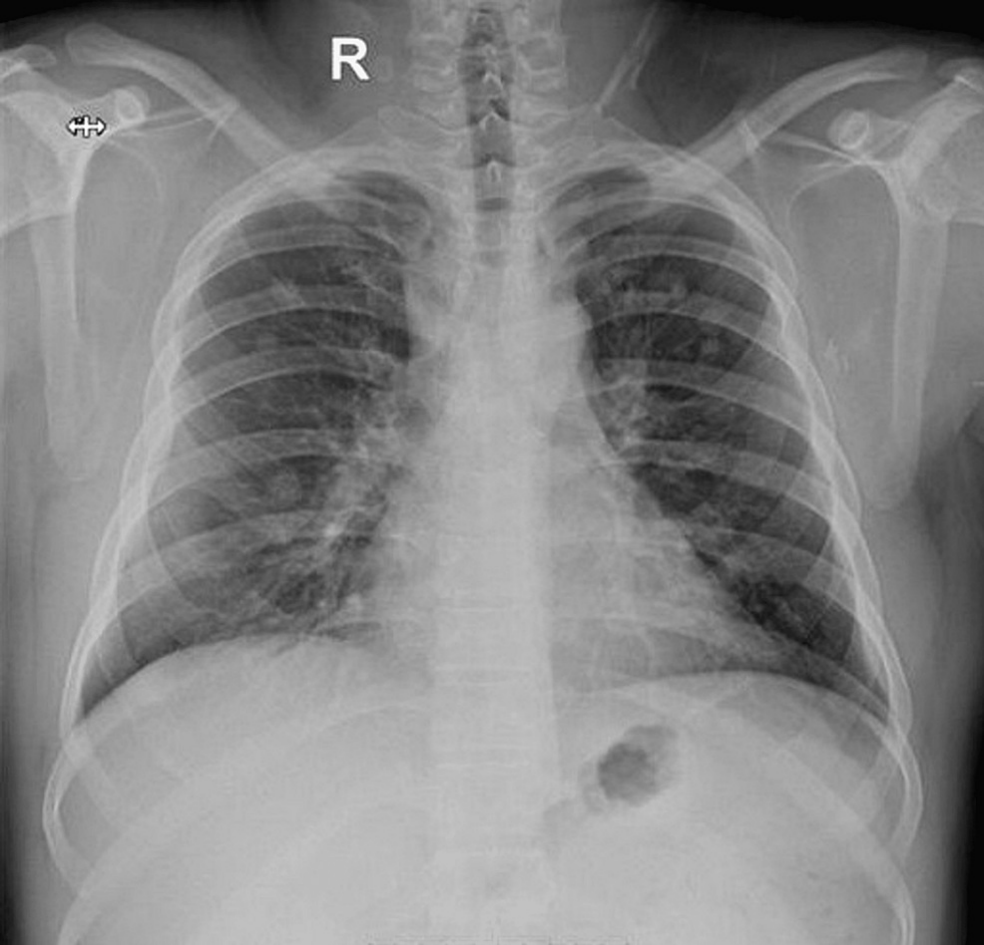
Chest X-ray done as a part of follow-up imaging. Interval development of multiple bilateral nodular lesions in the lungs.

During the regular follow-ups, three years after completing treatment, a chest X-ray (Figure 5) revealed the development of multiple bilateral nodular lesions in the lungs consistent with lung metastases.

This finding was confirmed on a CT scan, which also identified a metastatic adenocarcinoma in the right axillary lymph node. With the confirmation of an advanced disease, the patient was initiated on palliative chemotherapy, receiving six cycles of docetaxel and capecitabine. Restaging CT scan showed progressive disease with an increase in pulmonary metastasis, thoracic lymphadenopathy, multiple masses in the anterior chest wall, sternal metastasis, an anterior mediastinal mass, and right adrenal metastasis.

**Figure 6:**
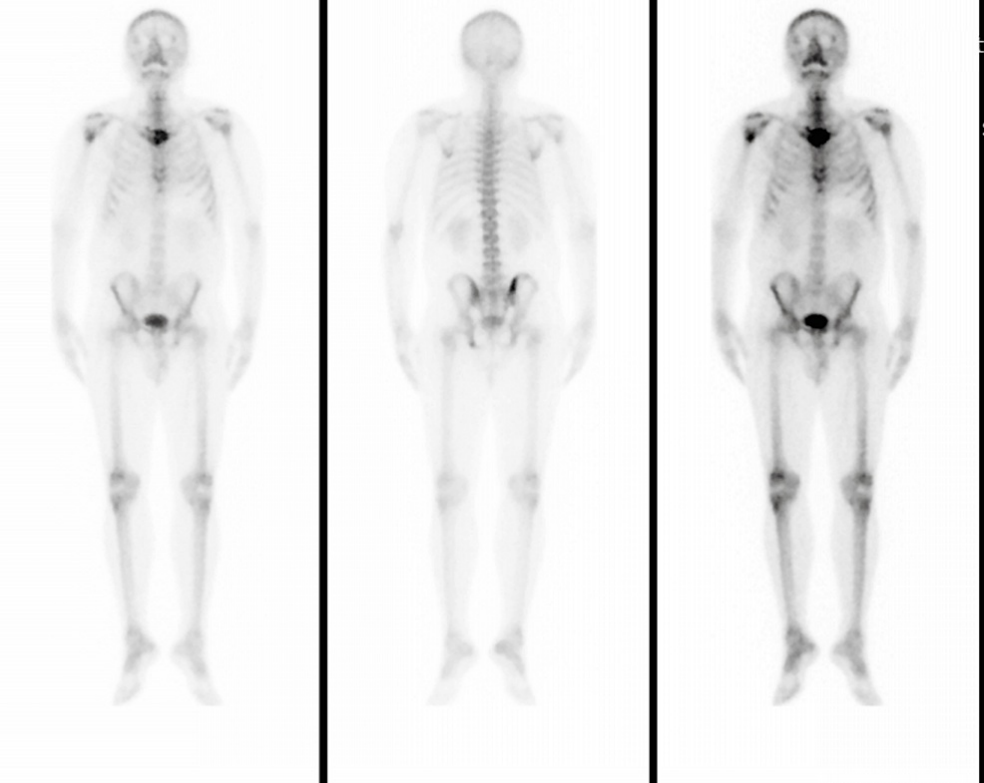
Bone scan showing evidence of metastases in the manubrium, sternum and right iliac bone, right humeral head, and left ischial body.

A bone scan revealed osseous metastases (Figure 6).

Meanwhile, the patient presented with seizures, and a magnetic resonance imaging (MRI) of the brain confirmed the presence of multiple bilateral supra- and infratentorial metastatic lesions. Whole brain radiation was administered for symptomatic control (2000 centigray (cGy) in five fractions).

At this point, the patient had advanced disease with metastases to the brain, lungs, thoracic lymph nodes, anterior chest wall, sternum, right humeral head, left ischial body, anterior mediastinum, and right adrenal gland. The patient was started on a cyclophosphamide, methotrexate, and fluorouracil (CMF) chemotherapy regimen.

Due to the development of excruciatingly painful locally recurrent disease on the anterior chest wall, the area was radiated with a single fraction of 800 cGy of electrons for local symptomatic control, which proved effective for pain control. 

Despite undergoing rigorous treatment, the patient's condition persistently deteriorated. Despite the valiant efforts to combat the disease, palliative care was initiated to manage his symptoms. Unfortunately, despite the patient's resilience, he ultimately succumbed to the illness and passed away.

## Discussion

Understanding the origin of metaplastic breast cancer remains an ongoing area of research. Studies suggest that metaplastic breast cancer may arise from the differentiation of breast epithelial cells into mesenchymal or spindle-like cells, resulting in a heterogeneous tumor with varying histological patterns. The etiology and specific risk factors for metaplastic breast cancer in male patients are still not well defined, further highlighting the need for continued research in this field. Despite its rarity, metaplastic breast cancer has gained attention due to its aggressive nature and limited treatment options. The current understanding of metaplastic breast cancer involves comprehensive molecular profiling and histological evaluation to identify potential therapeutic targets. However, due to the rarity of the disease, clinical trials and evidence-based treatment guidelines specific to metaplastic breast cancer in male patients are scarce [6,7].

It is crucial to recognize that men can also develop breast cancer, albeit at a significantly lower rate. Men diagnosed with breast carcinoma face unique challenges that often go unnoticed due to the prevailing stigmas associated with the disease. One of the primary challenges men face with breast carcinoma is delayed diagnosis. Because breast cancer is predominantly seen as a health issue among women, men and even healthcare professionals may overlook or misinterpret symptoms, leading to a delay in diagnosis. As a result, the cancer may progress to advanced stages, reducing the chances of successful treatment.

In the presented case, a young male in his early thirties presented with a lump in his left breast that had been present for more than six months. Thus, an educated man living in a big city with all the facilities presented six months after he first noticed the breast lump. The general public's awareness about male breast cancer is significantly lower compared to its female counterpart. The lack of awareness and education surrounding male breast cancer hampers early detection, prevention, and effective treatment. Men themselves may also be unaware that breast cancer can affect them, leading to missed opportunities for early intervention.

The psychological impact of a breast carcinoma diagnosis is profound for both men and women. However, men may face additional emotional challenges due to the rarity of the disease in their gender. Feelings of isolation, confusion, fear, and anxiety can be intensified when men find themselves in a predominantly female-oriented support network, and men may find dedicated women imaging centers and pink chemotherapy centers disturbing as the existing support networks and resources for breast cancer patients are primarily tailored toward women [8].

Men diagnosed with breast carcinoma often struggle to find support groups or organizations that specifically cater to their unique needs and concerns. This lack of support can exacerbate the feelings of isolation and prevent men from seeking necessary emotional and practical assistance [9].

Men with breast carcinoma may face specific treatment challenges. As the disease is less common in men, clinical trials and research studies often focus predominantly on female breast cancer. As a result, treatment options may not be as well studied or customized for men, leading to potential variations in treatment efficacy and outcomes. Breast cancer is commonly associated with women, and this gender stereotype can lead to stigmatization for men diagnosed with breast carcinoma. Society's expectations of masculinity may clash with the vulnerability and perceived femininity associated with a breast cancer diagnosis in men, which can lead to feelings of emasculation and shame [10].

Due to the relative rarity of male breast cancer, it receives less media coverage and public attention compared to female breast cancer. This lack of visibility contributes to the stigmatization of the disease, as it reinforces the notion that breast cancer is a women's issue. Men may feel invisible and unsupported, further perpetuating the stigma surrounding male breast carcinoma. Men diagnosed with breast cancer may experience social isolation due to the lack of understanding and awareness among their peers and communities. Friends, family, and coworkers may struggle to comprehend the challenges faced by men with breast carcinoma, leading to feelings of alienation and isolation. The stigma surrounding male breast cancer often discourages open and honest conversations about the disease. Men may find it difficult to discuss their diagnosis openly, fearing judgment or misunderstanding. This lack of dialogue hampers awareness and perpetuates the stigmatization of male breast carcinoma.

This case underscores the challenges faced in managing advanced metaplastic breast cancer in male patients. The rarity of the disease, limited treatment options, and lack of evidence-based guidelines make it difficult to achieve optimal outcomes. Furthermore, the social and psychological stigmas associated with male breast carcinoma add additional burdens to patients, hindering open discussion, seeking help, and sharing experiences.

Breast carcinoma in males, especially metaplastic breast carcinoma, is a rare and aggressive disease with distinct clinical and pathological characteristics. Late diagnosis, limited treatment options, and a lack of awareness among healthcare providers contribute to the challenges faced in managing this condition. The presented case highlights the urgent need for improved understanding, research, and targeted therapies for male breast carcinoma, as well as addressing the social and psychological stigmas associated with this condition to provide appropriate support and care for affected individuals.

## Conclusions

Breast carcinoma in males, particularly the rare subtype of metaplastic breast carcinoma characterized by its distinct histological features and aggressive nature, presents significant challenges in terms of diagnosis, treatment, and understanding. The case of a young male patient with metaplastic breast carcinoma demonstrated the potential for delayed diagnosis even in an educated individual living in an urban setting. The perception that breast cancer exclusively affects women leads to a lack of awareness, delayed diagnosis, and limited support resources for men. Men with breast carcinoma experience unique emotional challenges that require attention and support, including feelings of isolation, confusion, fear, and anxiety.

To address these issues, it is crucial to enhance education and awareness about male breast carcinoma among healthcare providers, the general public, and men themselves. Dedicated support networks and resources tailored to the needs of male patients should be established to provide a safe space for sharing experiences and seeking assistance. Continued research is necessary to improve our understanding of male breast carcinoma, develop effective treatment strategies, and address the psychosocial impact of the disease. By initiating discussions, promoting research, and addressing the stigmas associated with male breast carcinoma, we can strive for earlier diagnosis, improved treatment outcomes, and enhanced support for men facing this rare condition.
